# Potential Accumulative Effect of the Herbicide Glyphosate on Glyphosate-Tolerant Maize Rhizobacterial Communities over a Three-Year Cultivation Period

**DOI:** 10.1371/journal.pone.0027558

**Published:** 2011-11-11

**Authors:** Jorge Barriuso, Silvia Marín, Rafael P. Mellado

**Affiliations:** Departamento de Biotecnología Microbiana, Centro Nacional de Biotecnología (CNB-CSIC), Madrid, Spain; Instituto Butantan, Brazil

## Abstract

**Background:**

Glyphosate is a herbicide that is liable to be used in the extensive cultivation of glyphosate-tolerant cultivars. The potential accumulation of the relative effect of glyphosate on the rhizobacterial communities of glyphosate-tolerant maize has been monitored over a period of three years.

**Methodology/Principal Findings:**

The composition of rhizobacterial communities is known to vary with soil texture, hence, the analyses have been performed in two agricultural fields with a different soil texture. The accumulative effects of glyphosate have been monitored by means of high throughput DNA pyrosequencing of the bacterial DNA coding for the 16S rRNA hypervariable V6 region from rhizobacterial communities. The relative composition of the rhizobacterial communities does vary in each field over the three-year period. The overall distribution of the bacterial phyla seems to change from one year to the next similarly in the untreated and glyphosate-treated soils in both fields. The two methods used to estimate bacterial diversity offered consistent results and are equally suitable for diversity assessment.

**Conclusions/Significance:**

The glyphosate treatment during the three-year period of seasonal cultivation in two different fields did not seem to significantly change the maize rhizobacterial communities when compared to those of the untreated soil. This may be particularly relevant with respect to a potential authorisation to cultivate glyphosate-tolerant maize in the European Union.

## Introduction

Soil bacteria are known to affect plant growth and viability and thus play an important role in terrestrial agricultural ecosystems [1]. The routine application of herbicides to control the growth of undesirable weeds in cultivars is common practice in agricultural management, and alterations to the composition of rhizobacterial communities are likely to result from this and other agricultural practices such as fertilization or crop rotation [2], [3]. Glyphosate (2-(phosphonomethyl) glycine) is a less aggressive broad-spectrum systemic herbicide than those applied until now to control spontaneous weed growth, and glyphosate-tolerant crop varieties have been developed for maize, soybean, canola and cotton [4]. It is known that glyphosate absorbed in the leaves of glyphosate tolerant plants can alter root exudation and hence affect communities in the rhizosphere [5], [6], [7].

The effect of glyphosate on possible alterations to soil microorganisms from cultivated maize and other cultivars has recently been reviewed [6], [7], in additon to its possible effect on the emergence of weeds resistant to this herbicide [5].

Culture-independent nucleic acid-based methods have been used to characterise microbial communities. Among these, massive parallel pyrosequencing of the SSU rRNA V6 hypervariable region have proved to be very useful in the study of changes to the diversity of bacterial communities in many habitats, including soil [2], [8], [9], and in particular, to those of glyphosate-tolerant maize, where the post-emergence applied glyphosate was found to be environmentally less aggressive than the pre-emergence applied herbicide (Harness® GTZ) [10]. Since glyphosate is liable to be used generally for the extensive cultivation of glyphosate-tolerant cultivars, we monitored the potential accumulation of its relative effects on the rhizobacterial communities of glyphosate-tolerant maize by using massive parallel pyrosequencing over a three-year period in two different locations, as the composition of rhizobacterial communities varies with soil texture [11], [12]. To accomplish this, the structure of the bacterial communities was taxonomically analysed using MEGAN software [13] and phylogenetic distances were estimated with Fast UniFrac [14], [15], [16]. It has recently been reported that differences in diversity estimation may depend on the method used [17], [18], [19], therefore, two different methods were used to estimate bacterial diversity: a classic sequence alignment, MUSCLE [20] was followed by distance calculation, DNADIST [21] before using Mothur for OTUs' clustering [22], and the results were compared to those obtained with the one-step ESPRIT software package [19].

We analysed the potential effect of glyphosate on tolerant maize cultivation in two agricultural fields with soils of a different texture and found that the composition of the bacterial communities did vary, while the effect of the herbicide was very similar in the same bacterial groups. This may be particularly relevant as regards a potential authorisation to cultivate glyphosate-tolerant maize in the European Union.

## Results

Rhizobacterial DNA from the untreated soil or soil treated with glyphosate was amplified and subjected to pyrosequencing to characterise the herbicide effect on the maize rhizobacterial communities over a three-year period. The obtained sequences were grouped using the MEGAN program and NCBI taxonomy to generate the taxonomical trees. A week after the glyphosate treatment (first sampling time) and at the final stage of plant growth (final sampling time) DNA was extracted from the untreated and glyphosate-treated soil of each field. Tables 1 and S1 show the taxonomic breakdown as a result of sequencing the V6 region of the 16S rDNA genes extracted from field 1 at both sampling times, and show the distribution obtained from sequencing the 16S rDNA extracted from field 2 at both sampling times. The prominent phyla in most of the samples were *Proteobacteria*, *Actinobacteria* and *Acidobacteria*. The corresponding taxonomic trees obtained are included as Supplementary Information (Fig. S1).

**Table 1:** Taxonomic breakdown of the more relevant phyla from the twelve soils.

Field 1	2007		2008		2009	
	First sampling time					
Taxa	Untreated	Glyphosate	Untreated	Glyphosate	Untreated	Glyphosate
***Proteobacteria***	34.2	30.5	36.9	37.7	28.8	34.3
***Actinobacteria***	26.3	28.4	24.9	27.2	9.5	7.9
***Acidobacteria***	26.3	25.8	25.5	24.1	39.9	38.8
**Others**	13.2	15.3	12.7	11.0	21.8	19.0

	Final sampling time					
Taxa	Untreated	Glyphosate	Untreated	Glyphosate	Untreated	Glyphosate
***Proteobacteria***	32.6	35.7	35.8	33.4	25.1	28.1
***Actinobacteria***	18.1	16.7	22.4	24.4	10.0	12.2
***Acidobacteria***	27.5	26.7	25.4	24.8	41.7	43.5
**Others**	21.8	20.9	16.4	17.4	23.2	16.2

Field 2	2007		2008		2009	
	First sampling time					
Taxa	Untreated	Glyphosate	Untreated	Glyphosate	Untreated	Glyphosate
***Proteobacteria***	39.8	39.8	29.9	35.0	38.5	33.6
***Actinobacteria***	29.2	28.6	27.3	21.5	5.8	5.9
***Acidobacteria***	17.8	16.8	28.1	32.2	34.0	37.5
**Others**	13.2	14.8	14.7	11.3	21.7	23.0

	Final sampling time					
Taxa	Untreated	Glyphosate	Untreated	Glyphosate	Untreated	Glyphosate
***Proteobacteria***	33.2	35.0	30.3	31.0	32.9	33.2
***Actinobacteria***	10.6	8.1	29.5	29.4	4.8	7.5
***Acidobacteria***	38.1	31.8	24.1	26.4	36.0	32.4
**Others**	18.1	25.1	16.1	13.2	26.3	26.9

The percentages of *Proteobacteria*, *Actinobacteria* and *Acidobacteria* are indicated for field 1 and field 2 and do not include unassigned sequences.

A total 19.6% of the sequences analysed in the untreated soil of field 1 at the first sampling time in the first year remained unassigned. From the assigned sequences, 34.2% belong to the *Proteobacteria* phylum and 26.3% to the *Actinobacteria* phylum; another 26.3% are *Acidobacteria* and 13.2 % belong to other taxa. A very similar distribution of taxa (30.5% *Proteobacteria*, 28.4% *Actinobacteria,* 25.8% *Acidobacteria*, and 15.3% were other taxa) was found for the assigned sequences at an equivalent sampling time from the soil treated with glyphosate, where 19.3% of sequences remained unassigned. From the sequences analysed at the final sampling time, *Proteobacteria* and *Acidobacteria* were again prominent among the assigned sequences in the untreated soil (32.6% and 27.5%, respectively), while the presence of *Actinobacteria* diminished (18.1%), and 22.7% were unassigned sequences. At that particular sampling time the presence of *Actinobacteria* decreased further (16.7%) among the assigned sequences from the glyphosate treated soil, *Proteobacteria* (35.7%) and *Acidobacteria* (26.7%) being largely present, and 17.1% remained unassigned (Tables 1 and S1).

An equivalent pattern of phyla distribution was found at the first and final sampling times in field 2 soils treated or untreated with the herbicide, where the presence of *Actinobacteria* diminished at the final sampling time in both cases; this decline was slightly more conspicuous in the glyphosate treated soil (Tables 1 and S1).

In the second year, the distribution of taxa in the assigned sequences at the first sampling time of the untreated soil of field 1 was similar to that of the previous year: *Proteobacteria*, *Actinobacteria* and *Acidobacteria* were predominant and 15.7% remained unassigned. The same three phyla were also more abundant at the first sampling time of the glyphosate treated soil of the same field, where 19.6% were unassigned (Tables 1 and S1). At the final sampling time, *Proteobacteria*, *Actinobacteria* and *Acidobacteria* were again prominent among the assigned sequences in the untreated soil 1, where 22.7% were unassigned sequences, and this relative distribution did not change considerably at the same sampling time in the soil treated with glyphosate, where15.3% were unassigned sequences (Tables 1 and S1).

Once again, an equivalent presence of the predominant phyla was found at the first and final sampling times in field 2 soils treated or untreated with the herbicide, where the relative abundance of *Proteobacteria*, *Actinobacteria* and *Acidobacteria* remained comparatively similar in all cases (Tables 1 and S1).

At the first sampling time of field 1 untreated soil in the third year, the relative presence of the predominant phyla changed. *Proteobacteria* (28.8%) and *Acidobacteria* (39.9%) were still the most abundant phyla, the relative presence of *Actinobacteria* dropping to 9.5%. The relative presence of these three phyla was similar in the glyphosate treated soil, where 23.5% were unassigned sequences (Tables 1 and S1). At the final sampling time, the relative presence of *Proteobacteria* (25.1%) *Acidobacteria* (41.7%) and *Actinobacteria* (10.0%) seemed to remain the same in field 1 untreated soil (24.3% of unassigned sequences, which also appeared to be the case in the glyphosate treated soil at the same sampling time, where 23.1% remained unassigned (Tables 1 and S1). An equivalent result was achieved when the sequences obtained from field 2 glyphosate-treated and untreated soils at both sampling times were analysed, and just as in field 1 with the glyphosate-treated and untreated soil, the relative reduction in the number of *Actinobacteria* permitted the detection of other phyla (Tables 1 and S1).

**Figure 1:**
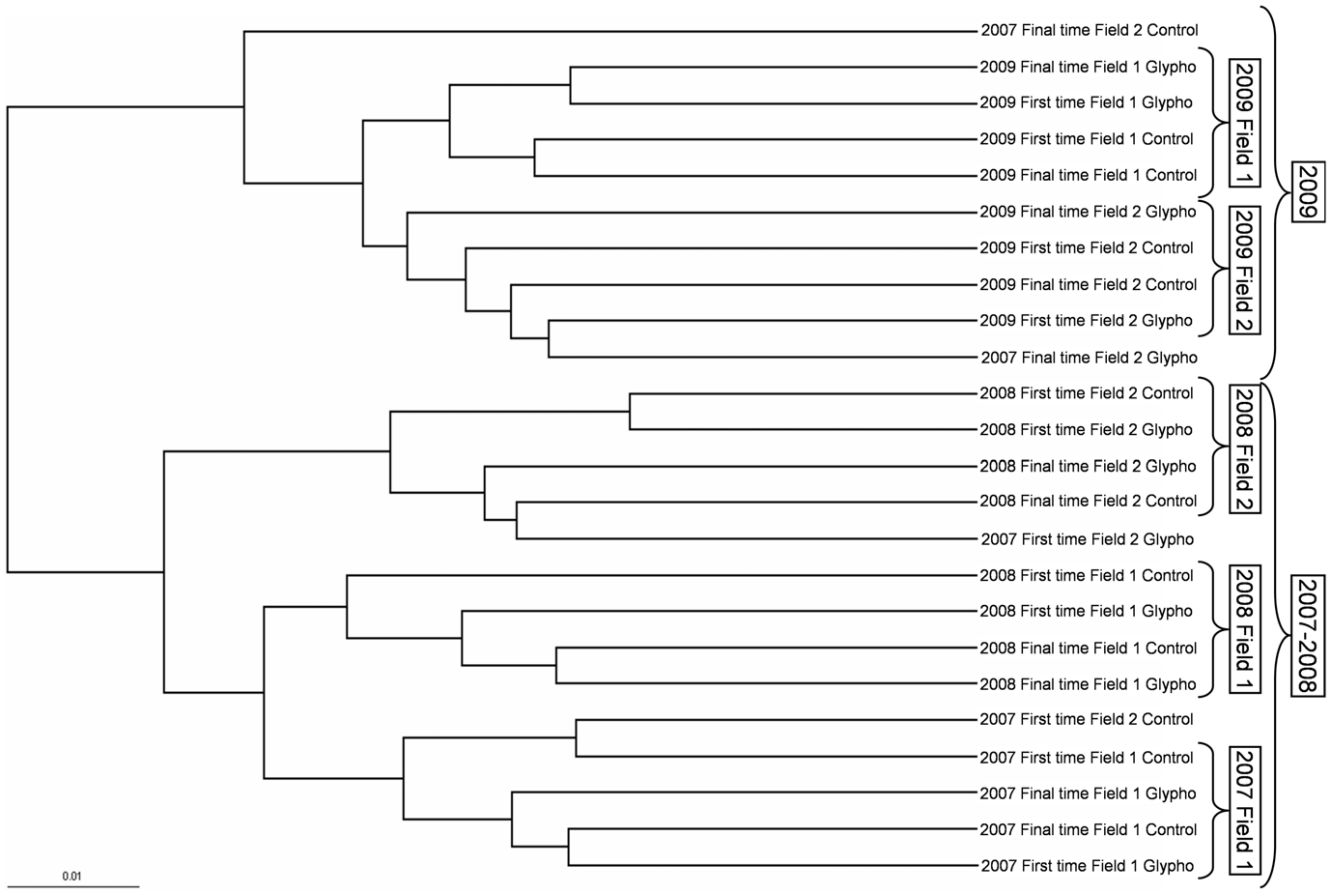
Fast UniFrac hierarchical clustering tree. Analysis of the different soil samples was carried out using normalized abundance weights.

Figure 1 shows the hierarchical clustering tree of samples based on the UniFrac metric. The rhizobacterial communities from 2007 and 2008 were more phylogenetically related, and the samples from 2009 are grouped in a separate branch of the tree, which also includes the 2007 two final samples from field 2. Fields are also grouped within each year. The UniFrac significance test showed no statistical differences between the untreated or treated samples within each year at any sampling time in both fields, except for field 2 samples from 2007. These results are in accordance with the taxonomic ones and show that there are no differences between the glyphosate-treated and untreated soils.

**Figure 2:**
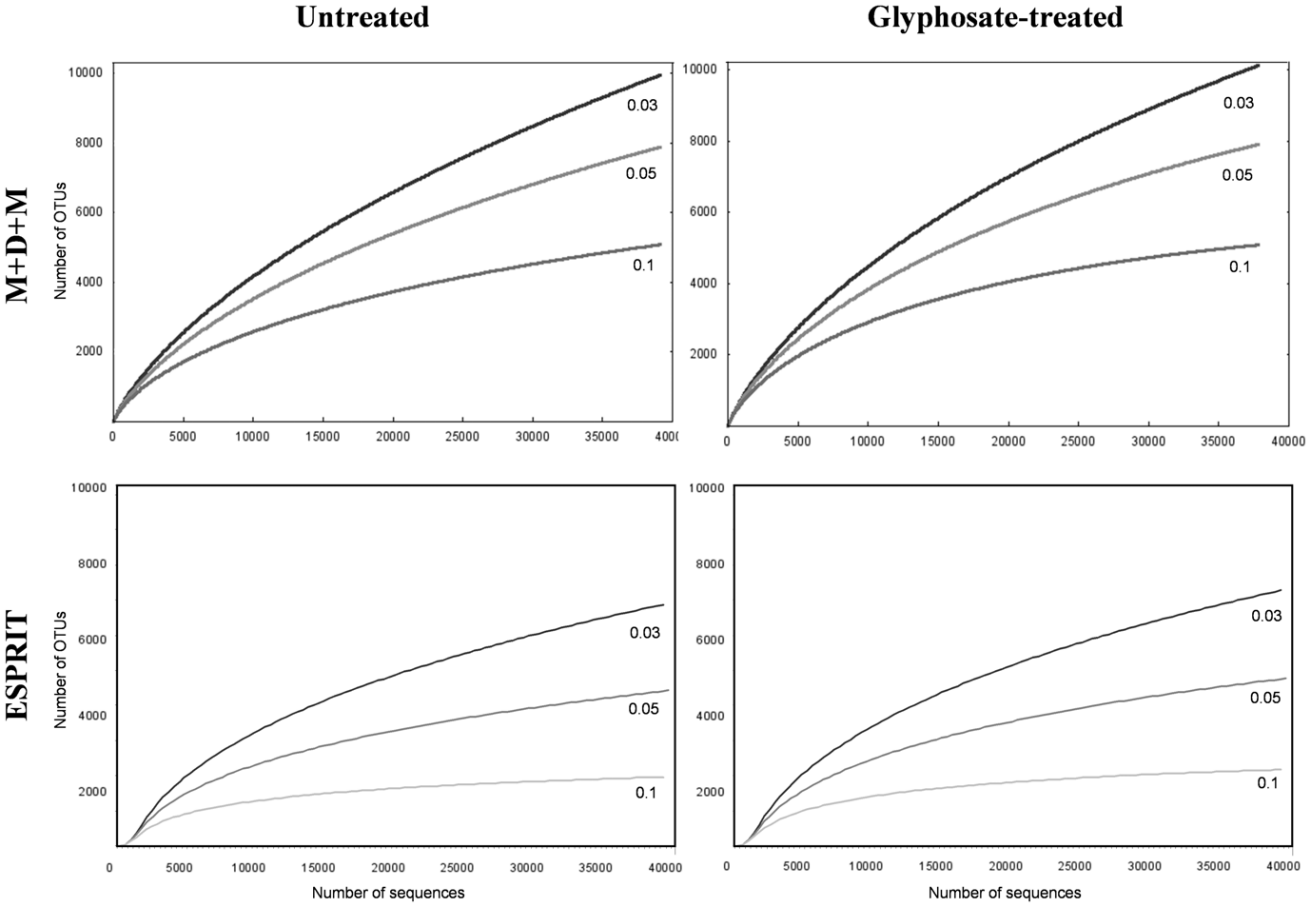
Rarefaction analysis. Rarefaction curves of representative samples taken in the first year at the final sampling time of glyphosate-treated and untreated soils from field 2 determined by using the MUSCLE, DNADIST and Mothur (M+D+M) combination or the ESPRIT program, are shown for OTUs with dissimilarities do not exceeding 3%, 5% or 10%. Rarefaction curves for the other samples showed a similar pattern.

When species richness was determined using the ACE and Chao1 estimators (Tables S2, S3 and S4), no significant differences seemed to be detected among the bacterial populations from the two fields during any of the three years. Table 2 shows a comparison of the OTUs analysis performed using MUSCLE for alignment, DNADIST for pairwise distance estimation and Mothur for clustering, in relation to the one conducted using the ESPRIT package only at 3% dissimilarity level. Table Tables S2, S3 and S4 contain the obtained data at 3%, 5% and 10% dissimilarity. The number of OTUs estimated when using the ESPRIT package was always lower, as previously reported [19]. Figure 2 shows the rarefaction curves for samples taken in the first year at the final sampling time from the untreated soil or soil treated with glyphosate from field 2 containing the largest number of sequences. The analysis was performed using the MUSCLE-DNADIST-Mothur combination and the ESPRIT package, and is a representative example of the rarefaction curves for the remaining samples (not shown). Rarefaction analyses for OTUs with dissimilarity not exceeding 3%, 5% or 10% showed slopes indicating that a considerable increase in the number of sequences would not lead to higher estimates of total diversity, especially when relative large genetic distances (5% or 10% dissimilarity) define similarity groups, particularly in the case of ESPRIT (Fig. 2), while a larger number of sequences would be needed to obtain a more accurate estimate of total bacterial diversity, when dealing with distances at 3% dissimilarity that define shorter genetic distances.

**Table 2:** Similarity-based OTUs estimates at a 3% dissimilarity level.

Field 1	2007				2008				2009			
	First sampling time				First sampling time				First sampling time			
	Untreated		Glyphosate		Untreated		Glyphosate		Untreated		Glyphosate	
	M+D+M	ESPRIT	M+D+M	ESPRIT	M+D+M	ESPRIT	M+D+M	ESPRIT	M+D+M	ESPRIT	M+D+M	ESPRIT
**OTUs**	631	597	632	571	604	587	655	588	756	622	734	621

	Final sampling time				Final sampling time				Final sampling time			
	Untreated		Glyphosate		Untreated		Glyphosate		Untreated		Glyphosate	
	M+D+M	ESPRIT	M+D+M	ESPRIT	M+D+M	ESPRIT	M+D+M	ESPRIT	M+D+M	ESPRIT	M+D+M	ESPRIT
**OTUs**	671	592	569	575	622	522	566	532	753	589	703	582

Field 2	2007				2008				2009			
	First sampling time				First sampling time				First sampling time			
	Untreated		Glyphosate		Untreated		Glyphosate		Untreated		Glyphosate	
	M+D+M	ESPRIT	M+D+M	ESPRIT	M+D+M	ESPRIT	M+D+M	ESPRIT	M+D+M	ESPRIT	M+D+M	ESPRIT
**OTUs**	597	585	687	622	647	629	604	607	699	624	761	634

	Final sampling time				Final sampling time				Final sampling time			
	Untreated		Glyphosate		Untreated		Glyphosate		Untreated		Glyphosate	
	M+D+M	ESPRIT	M+D+M	ESPRIT	M+D+M	ESPRIT	M+D+M	ESPRIT	M+D+M	ESPRIT	M+D+M	ESPRIT
**OTUs**	578	481	603	499	737	634	659	592	642	533	674	543

The species richness estimates were determined by using the MUSCLE, DNADIST and Mothur (M+D+M) combination or the ESPRIT program, as described in Materials and Methods.

## Discussion

The potential effect of the glyphosate treatment on glyphosate tolerant maize (event NK603) has previously been analysed by pyrosequencing the hypervariable V6 region of a large number of 16S rRNA sequences, hence revealing that glyphosate, is environmentally less aggressive than other herbicides [10]. In this study the potentially cumulative effect of glyphosate on glyphosate-tolerant maize has been monitored during the continuous cultivation of NK603 maize for three consecutive years in two different agricultural fields. The soil texture of the two fields was slightly different; field 1 appeared to be sandy-loam while field 2 appeared to be loam. Three phyla seemed predominant in both fields: *Proteobacteria*, *Actinobacteria* and *Acidobacteria*. The overall distribution of these three phyla seemed to change from year to year similarly at both sampling times in the untreated and glyphosate treated soils in both fields throughout the first two years of the assay (Tables 1 and S1). In the third year, *Actinobacteria* no longer seemed to be predominant in any of the soils and fields, yet the relative rhizobacterial composition seemed to remain the same in the untreated and glyphosate-treated soils (Tables 1 and S1). The reason why the structure of the rhizobacterial community changed in the third year in both fields is still unknown, however, we believe it may be related to a particular climate change, comprising a lenghthier period of rainfall during the winter/spring season between the second and third year, where almost three times more rain had accumulated than during the same period in previous years (http://clima.meteored.com). These results are in accordance with earlier findings [10], where a mild glyphosate effect was observed on the maize rhizobacterial community in the first sampling time and the rhizobacterial population was able to fully recover from it throughout plant growth [10]. Seven bacterial phyla have been found to be predominant in rhizospheres: *Proteobacteria, Actinobacteria, Acidobacteria, Verrucomicrobia, Planctmycetes*, *Bacteriodetes* and *Firmicutes* [23], therefore, it was not surprising to see *Acidobacteria* and *Verrucomicrobia* compensating the absence of *Actinobacteria* observed in the third year in both soils. *Acidobacteria* are commonly detected in soils [24] and it has been suggested that members of this phylum are likely to play a relevant role in conducting processes in terrestrial ecosystems [25].

Although each field had its own texture, the fields were relatively close to each other and were able to share the same climatological fluctuations; moreover, the phylogenetic analysis confirmed the taxonomic findings. Thus, the trends observed in the taxonomic analysis of the two fields (Tables 1 and S1) were confirmed by the UniFrac statistical phylogenetic analysis which did not show significant differences among the samples within each year except for field 2 samples from 2007, yet unidentified environmental factors could eventually explain the 2007 samples from field 2 clustering with those of 2009 (Fig. 1). The relative changes to *Actinobacteria* composition during plant growth in some of the herbicide-treated or untreated soils were not significant (Fig.1).

These results are in agreement with those obtained by other authors on maize [26] who observed no changes in functional bacterial communities and fungi from the rhizosphere of maize plants treated with glyphosate when compared to the untreated control, rhzobacterial fluctuations being on account of seasonal changes rather than herbicide action. This low level of glyphosate aggressiveness is most likely due to its strong capacity to bind to normal soil particles (high Kd), which has also been described before for other crops [27], [28] but apparently is in contrast to the effects observed for soybean rhizospheres [29]. However, these results may not be strictly comparable with those of maize as they were obtained in a greenhouse study (not in an open field) and focussed on some particular groups of culturable root-colonizer bacteria and fungi.

For diversity estimation, the MUSCLE algorithm was chosen to align the sequences, combined with the DNADIST program, with Jukes-Cantor correction to estimate pairwise distances and Mothur for the data clustering. The data obtained were compared with the analysis of the ESPRIT program, which does not require a complete alignment of all the sequences. The two methods showed no statistically significant differences in the estimated bacterial diversity, which was similar in both fields in the untreated or glyphosate-treated soils (Tables 2 and S2, S3 and S4). Although the two methods used to analyse bacterial diversity showed some differences, both of these offered consistent results when estimating changes in the bacterial communities, although a lower OTUs estimation was revealed when using ESPRIT, as previously reported [18], [19], [30]. Despite this, the two methods produced qualitatively equivalent results, and therefore, are equally suitable for assessing the diversity of rhizobacterial communities. Consequently, the rarefaction curves obtained using the MUSCLE-DNADIST-Mothur combination showed a steeper incline than those obtained using ESPRIT (Fig. 2), as the first combination rendered a higher number of OTUS, suggesting that regardless of the method used, a greater number of sequences will be needed to determine the bacterial diversity of the soil. In any event, the coverage of overall diversity was not the aim of this study.

Taken together, the results obtained show that the glyphosate treatment does not seem to dramatically change the maize rhizobacterial communities, when compared to those of the untreated soil over a three-year period of seasonal cultivation in two different fields. The differences perceived in the composition of the rhizobacterial communities were most likely due to the fluctuations in climate and affected the untreated and the herbicide-treated soils almost equally, probably reflecting the need to perform soil monitoring analyses of rhizobacterial communities for periods of more than three years to assess the potential impact on non-target organisms such as the rhizobacteria of herbicide-tolerant plant varieties, in the event of these being authorised for cultivation in soils of the European Union.

## Materials and Methods

### Plant materials and sampling

Glyphosate-tolerant maize, event NK603, variety DKC6452RR, containing a glyphosate-tolerant EPSPS enzyme (5-enolpyruvylshikimate-3-phosphate synthase) derived from the CP4 strain of the common soil bacterium *Agrobacterium* sp. [31] was grown in two different commercial maize fields located in Lérida, Spain (field 1, N41° 37′ 48″ E0° 40′ 12″ and field 2, N41° 40′ 47″ E0° 42′ 0″), where no NK603 maize was grown before. Current agricultural practises were maintained during the three years of cultivation. An equivalent number of plants were treated in post-emergence with glyphosate, (Roundup®Plus containing 360 g/l glyphosate as isopropylamine salt; 0.72 kg/ha) or not treated with herbicide at all (control soil). The surface of each experimental field was 40 m^2^. Maize NK603 and both herbicides were from Monsanto.

The glyphosate was applied approximately two months after seeding and the plants were harvested from the herbicide-treated and untreated soils at two different growth stages: 7 days after glyphosate application (first sampling time), when the plants were around 10 cm tall and had 2–3 leaves, and just before crop harvesting at final growth (final sampling time). Roots and adhered soil measuring approximately 2 mm or less in diameter were separated from the bulk soil by gently shaking the root system. The term “rhizosphere” describes the carefully separated soil adhered to these roots. Given the small size of the maize fields, these were divided into subplots, and 3 samples were taken from each subplot at the time of collection. A total of 9 subplots were collected from each maize field at every collection and an equal amount of soil from each of the 27 samples was pooled in all cases. The experiments were performed on NK603 maize during development throughout 2007, 2008 and 2009.

### Texture and chemical properties of the soils

The agricultural soil texture of field 1 appears to be sandy-loam containing 14% clay, 29% silt and 57% sand, while the soil texture of field 2 appears to be loam containing 10% clay, 42% silt and 48% sand, as determined by Agriquem S.L. (Seville, Spain). Organic matter (OM) was measured every year in both fields: values for field 1 were 3.14%, 3.44% and 3.83% for 2007, 2008 and 2009 respectively, and for field 2, 3.26%, 3.47% and 3.77% for 2007, 2008 and 2009 respectively. Adsorption coefficient (Kd) values were estimated based on Koc values (Koc = 100Kd/%OC) [32], as detailed in the PPDB (pesticide properties database) web site (http://sitem.herts.ac.uk/aeru/footprint/). Considering that %OC = %OM/1.7, the estimated glyphosate Kd values for field 1 were 400.79, 439.09 and 488.87 for 2007, 2008 and 2009 respectively, and for field 2 were 416.11, 442.91 and 481.21 for 2007, 2008 and 2009 respectively.

### DNA extraction, PCR amplification and pyrosequencing

Rhizospheres from each of the different collection times were pooled and the soil was subjected to three independent DNA extractions using the PowerMax Soil DNA kits (MO Bio Laboratories Inc., USA), following instructions from the supplier. Soil DNA from each of the three independent extractions was used as template for PCR amplification of the V6 hypervariable region of the 16S rRNA gene. The oligonucleotide design included 454 Life Science's Titanium A or B sequencing adaptors fused to the 5′ end of primer 967F (5′- CAACGCGAAGAACCTTACC –3′) and 1046R (5′- CGACAGCCATGCANCACCT –3′), where a MID (Multiplex Identifier) was included immediately preceding the V6 specific primer. A different MID was included for each soil sample. Table S5 shows the MID used for each sample and the number of sequences obtained before filtering; sequence numbers resulted from the accumulation of several runs. PCR amplification was performed by incubation at 95°C for 5 min, followed by 30 cycles of incubation at 95°C (30 sec), 63°C (45 sec) and 72°C (1 min), with a final extension cycle of 5 min at 72°C. The amplified DNA resulting from the three independent PCR reactions for each DNA template preparation was pooled and cleaned (Illustra GFX PCR DNA purification kit, GE Healthcare), checked with the Bioanalyser 2100 (Agilent technologies), quantified with Quant-IT-picogreen (Invitrogen) and used to make the single strands on beads, as required for 454 Titanium pyrosequencing [33]. A total of 321729 sequences were obtained from the different sampling times in both fields and the sequences were deposited in the NCBI sequence reads archives (accession number SRX063207).

For taxonomical purposes, the 454 reads for the V6 regions of each soil were filtered to eliminate the short sequences that account for 50% of all pyrosequencing errors [17] and then compared with the SSU rRNA SILVA database version 92 [34] using BLASTN. Files containing the 25 best matches for each of the 454 determined sequences were used as input to generate the corresponding taxonomic trees by means of the MEGAN 2.0 program [13].

Fast UniFrac (http://bmf2.colorado.edu/fastunifrac) [14], [15], [16] was used to carry out a hierarchical clustering of the samples based on their phylogenetic distances. The analysis was performed with the Greengenes core as a reference phylogenetic tree, Jackknife supporting values and calculated using normalized abundance weights. The Fast UniFrac significance test was used to assess the existence of statistically significant differences among the 16S rDNA sequences from each soil sample, based on their phylogenetic distances.

To assess taxonomic-independent diversity, an equal number of sequences (1000) were randomly selected from each soil, and the selected pools of sequences were aligned using the MUSCLE program [20]. The resulting alignment was transformed into a distance matrix using the DNADIST program (PHYLIP software package version 3.68 with Jukes-Cantor correction) [21], which served as input for Mothur [22] to cluster tags into OTUs (operational taxonomic units), generate rarefaction curves and calculate species diversity with the Chao1 and ACE estimators. The obtained results were compared with those generated by the ESPRIT software (using the *–*f parameter), which avoids the initial multiple sequence alignment step by applying an efficient k-tuple based distance filter, subsequently aligning the sequences using the Needleman-Wunsch method and computing pairwise distances using the quickdist algorithm [19], hence, a comparison is made of the efficiency of both methods and species diversity estimation. Regarding species richness estimation, confidence intervals were calculated with DOTUR and set to 95% for all Chao1 and ACE data, with the exception of those generated by ESPRIT. The statistical significance of the observed Chao1 differences was determined by the Tukey-Kramer test.

## Supporting Information

Figure S1 **Taxonomic trees.** Taxonomic trees resulting from pyrosequencing the V6 region of the 16S rDNA extracted from each field at the indicated sampling times are shown. The size of the dots reflects the relative amount of taxa assigned to each particular node.
(TIF)

Table S1 **Taxonomic breakdown of the more relevant phyla from the twelve soils.** The percentages of *Proteobacteria*, *Actinobacteria, Bacteroidetes, Verrucomicrobia, Planctomycetes, Chloroflexi, Acidobacteria, Nitrospira, Gemmatimonadetes and Firmicutes* are indicated for field 1 and field 2 and do not include unassigned sequences.
(PDF)

Table S2 **Similarity-based OTUs and species richness estimates at a 3%, 5% and 10% dissimilarity level for samples from 2007.** The species richness estimates were determined by using the MUSCLE, DNADIST and Mothur (M+D+M) combination or the ESPRIT program, as described in Materials and Methods.
(PDF)

Table S3 **Similarity-based OTUs and species richness estimates at a 3%, 5% and 10% dissimilarity level for the samples from 2008.** The species richness estimates were determined by using the MUSCLE, DNADIST and Mothur (M+D+M) combination or the ESPRIT program, as described in Materials and Methods.
(PDF)

Table S4 **Similarity-based OTUs and species richness estimates at a 3%, 5% and 10% dissimilarity level for the samples from 2009.** The species richness estimates were determined by using the MUSCLE, DNADIST and Mothur (M+D+M) combination or the ESPRIT program, as described in Materials and Methods.
(PDF)

Table S5 **Multiplex identifiers (MIDs).** The Multiplex identifiers (MIDs) used for pyrosequencing the different samples are shown.
(PDF)
