# Supplementary figures and images for: Potential Accumulative Effect of the Herbicide Glyphosate on Glyphosate-Tolerant Maize Rhizobacterial Communities over a Three-Year Cultivation Period
Source: PLoS One. 2011 Nov 11;6(11):e27558. doi: 10.1371/journal.pone.0027558 (PMC3214082; doi:10.1371/journal.pone.0027558)

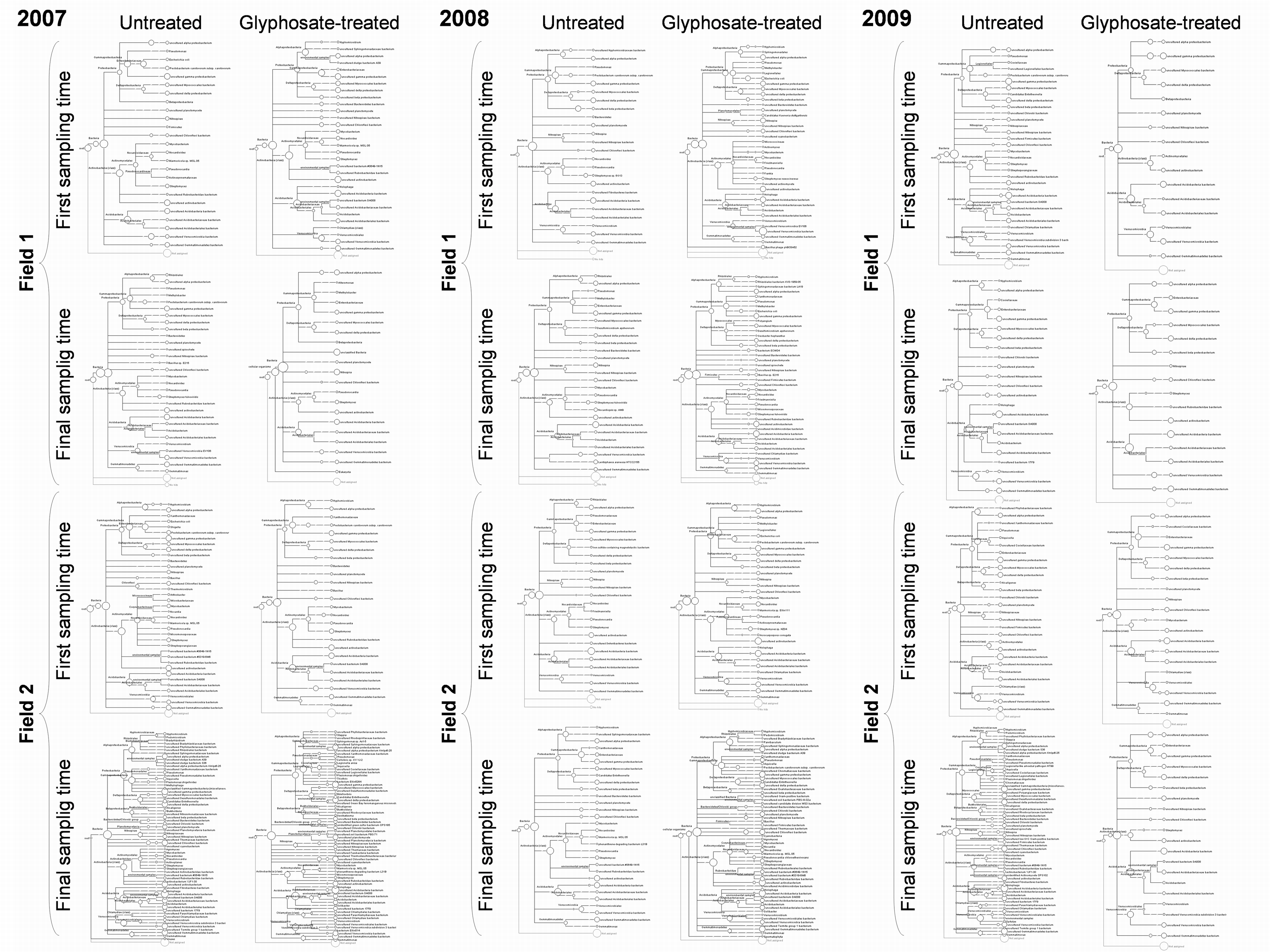

Supplement: Figure S1 — Taxonomic trees. Taxonomic trees resulting from pyrosequencing the V6 region of the 16S rDNA extracted from each field at the indicated sampling times are shown. The size of the dots reflects the relative amount of taxa assigned to each particular node. (TIF) [file pone.0027558.s001.tif]
