# Supplementary material for: Potential Accumulative Effect of the Herbicide Glyphosate on Glyphosate-Tolerant Maize Rhizobacterial Communities over a Three-Year Cultivation Period
Source: PLoS One. 2011 Nov 11;6(11):e27558. doi: 10.1371/journal.pone.0027558 (PMC3214082; doi:10.1371/journal.pone.0027558)
Supplement: Table S1 — Taxonomic breakdown of the more relevant phyla from the twelve soils. The percentages of Proteobacteria, Actinobacteria, Bacteroidetes, Verrucomicrobia, Planctomycetes, Chloroflexi, Acidobacteria, Nitrospira, Gemmatimonadetes and Firmicutes are indicated for field 1 and field 2 and do not include unassigned sequences. (PDF) [file pone.0027558.s002.pdf]

Table S1. Taxonomic breakdown of the more relevant phyla from the twelve soils.

**Field 1**	**2007**		**2008**		**2009**	
			**First sampling time**			
**Taxa**	**Untreated**	**Glyphosate**	**Untreated**	**Glyphosate**	**Untreated**	**Glyphosate**
Proteobacteria	34.2	30.5	36.9	37.7	28.8	34.3
Actinobacteria	26.3	28.4	24.9	27.2	9.5	7.9
Bacteroidetes	0.0	0.4	0.0	0.0	0.2	0.0
Verrucomicrobia	6.4	7.1	5.6	4.1	11.9	9.5
Planctomycetes	0.5	1.2	1.2	0.7	1.1	2.0
Chloroflexi	1.2	2.2	0.9	1.5	1.6	1.7
Acidobacteria	26.3	25.8	25.5	24.1	39.9	38.8
Nitrospira	1.2	0.9	2.5	1.2	2.1	1.8
Gemmatimonadetes	3.6	3.3	2.1	3.3	4.4	4.0
Firmicutes	0.3	0.2	0.4	0.2	0.5	0.0
			**Final sampling time**			
**Taxa**	**Untreated**	**Glyphosate**	**Untreated**	**Glyphosate**	**Untreated**	**Glyphosate**
Proteobacteria	32.6	35.7	35.8	33.4	25.1	28.1
Actinobacteria	18.1	16.7	22.4	24.4	10.0	12.2
Bacteroidetes	0.0	0.0	0.0	0.0	0.4	0.0
Verrucomicrobia	10.1	12.5	9.1	9.2	13.6	6.6
Planctomycetes	1.0	0.7	0.8	0.9	1.5	0.0
Chloroflexi	1.5	1.6	1.4	1.8	0.9	2.0
Acidobacteria	27.5	26.7	25.4	24.8	41.7	43.5
Nitrospira	1.5	1.2	2.3	1.6	1.6	0.7
Gemmatimonadetes	6.9	3.7	2.3	4.0	5.1	6.8
Firmicutes	0.5	1.0	0.0	0.0	0.0	0.0

**Field 2**	**2007**		**2008**		**2009**	
			**First sampling time**			
**Taxa**	**Untreated**	**Glyphosate**	**Untreated**	**Glyphosate**	**Untreated**	**Glyphosate**
Proteobacteria	39.8	39.8	29.9	35.0	38.5	33.6
Actinobacteria	29.2	28.6	27.3	21.5	5.8	5.9
Bacteroidetes	0.2	0.4	0.0	0.0	0.3	0.0
Verrucomicrobia	5.2	5.1	6.2	4.3	12.2	12.1
Planctomycetes	0.6	0.7	1.3	0.7	1.5	0.3
Chloroflexi	2.1	1.8	1.0	1.5	1.1	2.9
Acidobacteria	17.8	16.8	28.1	32.2	34.0	37.5
Nitrospira	1.1	1.9	2.8	1.3	2.3	1.1
Gemmatimonadetes	3.7	4.5	2.4	3.3	4.0	6.6
Firmicutes	0.3	0.4	1.0	0.2	0.3	0.0
			**Final sampling time**			
**Taxa**	**Untreated**	**Glyphosate**	**Untreated**	**Glyphosate**	**Untreated**	**Glyphosate**
Proteobacteria	33.2	35.0	30.3	31.0	32.9	33.2
Actinobacteria	10.6	8.1	29.5	29.4	4.8	7.5
Bacteroidetes	0.5	1.5	0.3	0.2	0.5	0.2
Verrucomicrobia	7.7	10.4	5.6	3.8	12.0	13.0
Planctomycetes	1.2	2.2	0.6	0.6	1.2	5.0
Chloroflexi	2.5	3.1	2.7	3.3	2.7	2.6
Acidobacteria	38.1	31.8	24.1	26.4	36.0	32.4
Nitrospira	1.7	1.5	1.6	0.9	1.8	1.5
Gemmatimonadetes	4.4	6.3	5.1	4.2	7.7	4.6
Firmicutes	0.1	0.1	0.2	0.2	0.4	0.0

The percentages of *Proteobacteria, Actinobacteria, Bacteroidetes, Verrucomicrobia, Planctomycetes, Chloroflexi, Acidobacteria, Nitrospira, Gemmatimonadetes* and *Firmicutes* are indicated for field 1 and field 2 and do not include unassigned sequences.
