# Supplementary material for: Potential Accumulative Effect of the Herbicide Glyphosate on Glyphosate-Tolerant Maize Rhizobacterial Communities over a Three-Year Cultivation Period
Source: PLoS One. 2011 Nov 11;6(11):e27558. doi: 10.1371/journal.pone.0027558 (PMC3214082; doi:10.1371/journal.pone.0027558)
Supplement: Table S2 — Similarity-based OTUs and species richness estimates at a 3%, 5% and 10% dissimilarity level for samples from 2007. The species richness estimates were determined by using the MUSCLE, DNADIST and Mothur (M+D+M) combination or the ESPRIT program, as described in Materials and Methods. (PDF) [file pone.0027558.s003.pdf]

Table S2.1. Similarity-based OTUs and species richness estimates at a 3%, 5% and 10% dissimilarity level for the samples from 2007

**Field 1**	**2007**											
	**First sampling time**											
	**Untreated**						**Glyphosate**					
	**M+D+M**			**ESPRIT**			**M+D+M**			**ESPRIT**		
	3%	5%	10%	3%	5%	10%	3%	5%	10%	3%	5%	10%
**OTUs**	631	524	306	597	435	195	632	505	300	571	413	186
**ACE**	1555±242	1024±133	386±34	1393	765	226	1371±183	843±93	391±37	1190	693	206
**Chao1**	1497±237	1042±162	391±44	1436±242	751±112	213±16	1281±175	802±96	369±36	1127±162	669±95	199±13
	**Final sampling time**											
	**Untreated**						**Glyphosate**					
	**M+D+M**			**ESPRIT**			**M+D+M**			**ESPRIT**		
	3%	5%	10%	3%	5%	10%	3%	5%	10%	3%	5%	10%
**OTUs**	671	585	419	592	432	213	569	484	348	575	409	226
**ACE**	1503±208	1147±143	704±84	1214	723	246	1262±179	1036±151	597±82	1399	853	293
**Chao1**	1413±192	1122±153	677±95	1128±150	647±77	241±21	1217±190	1006±171	570±91	1238±191	786±135	267±26

**Field 2**	**2007**											
	**First sampling time**											
	**Untreated**						**Glyphosate**					
	**M+D+M**			**ESPRIT**			**M+D+M**			**ESPRIT**		
	3%	5%	10%	3%	5%	10%	3%	5%	10%	3%	5%	10%
**OTUs**	597	478	282	585	413	184	687	576	405	622	445	224
**ACE**	1629±273	964±133	363±35	1278	1278	1278	2006±341	1254±178	718±95	1491	760	259
**Chao1**	1476±248	907±140	354±39	1301±204	672±93	196±12	1840±301	1168±169	677±100	1397±214	734±101	245±17
	**Final sampling time**											
	**Untreated**						**Glyphosate**					
	**M+D+M**			**ESPRIT**			**M+D+M**			**ESPRIT**		
	3%	5%	10%	3%	5%	10%	3%	5%	10%	3%	5%	10%
**OTUs**	578	494	367	481	342	184	603	507	328	499	377	205
**ACE**	1379±205	1014±141	716±109	977	678	257	1488±218	961±124	451±45	896	586	244
**Chao1**	1389±240	1062±186	624±99	986±169	675±135	251±43	1255±180	879±117	414±41	867±117	552±68	238±24

The species richness estimates were determined by using the combination MUSCLE, DNADIST and Mothur (M+D+M) or the ESPRIT programs as described in Materials and methods.
