# Supplementary material for: Potential Accumulative Effect of the Herbicide Glyphosate on Glyphosate-Tolerant Maize Rhizobacterial Communities over a Three-Year Cultivation Period
Source: PLoS One. 2011 Nov 11;6(11):e27558. doi: 10.1371/journal.pone.0027558 (PMC3214082; doi:10.1371/journal.pone.0027558)
Supplement: Table S3 — Similarity-based OTUs and species richness estimates at a 3%, 5% and 10% dissimilarity level for the samples from 2008. The species richness estimates were determined by using the MUSCLE, DNADIST and Mothur (M+D+M) combination or the ESPRIT program, as described in Materials and Methods. (PDF) [file pone.0027558.s004.pdf]

Table S2.2. Similarity-based OTUs and species richness estimates at a 3%, 5% and 10% dissimilarity level for the samples from 2008

**Field 1**	**2008**											
	**First sampling time**											
	**Untreated**						**Glyphosate**					
	**M+D+M**			**ESPRIT**			**M+D+M**			**ESPRIT**		
	3%	5%	10%	3%	5%	10%	3%	5%	10%	3%	5%	10%
**OTUs**	604	524	386	587	426	222	655	554	380	588	416	185
**ACE**	1633±255	1280±197	761±115	1482	764	256	1801±294	1178±165	607±73	1245	682	198
**Chao1**	1403±223	1114±180	662±102	1232±181	711±100	240±15	1681±278	1121±168	595±84	1250±191	664±93	190±8
	**Final sampling time**											
	**Untreated**						**Glyphosate**					
	**M+D+M**			**ESPRIT**			**M+D+M**			**ESPRIT**		
	3%	5%	10%	3%	5%	10%	3%	5%	10%	3%	5%	10%
**OTUs**	622	521	373	522	357	195	566	494	367	532	336	199
**ACE**	1675±270	1274±196	772±123	1005	752	302	2571±550	1725±340	812±143	1097	735	318
**Chao1**	1438±223	1094±175	703±126	1015±154	716±142	312±68	2123±487	1360±273	701±123	1018±153	675±130	311±63

**Field 2**	**2008**											
	**First sampling time**											
	**Untreated**						**Glyphosate**					
	**M+D+M**			**ESPRIT**			**M+D+M**			**ESPRIT**		
	3%	5%	10%	3%	5%	10%	3%	5%	10%	3%	5%	10%
**OTUs**	647	525	393	629	458	209	604	487	380	607	473	226
**ACE**	1919±286	1145±138	608±69	1400	841	229	1759±192	1060±159	587±112	580	380	243
**Chao1**	1781±278	1074±142	608±84	1326±188	775±106	219±10	1521±172	978±102	524±107	594±77	433±102	211±68
	**Final sampling time**											
	**Untreated**						**Glyphosate**					
	**M+D+M**			**ESPRIT**			**M+D+M**			**ESPRIT**		
	3%	5%	10%	3%	5%	10%	3%	5%	10%	3%	5%	10%
**OTUs**	737	620	382	634	472	215	659	541	354	592	435	210
**ACE**	2199±362	1461±221	526±49	1439	853	234	1452±189	1057±134	556±68	1173	680	239
**Chao1**	2182±368	1361±204	480±42	1401±209	850±128	225±10	1464±213	1030±149	588±99	1127±152	661±83	240±24

The species richness estimates were determined by using the combination MUSCLE, DNADIST and Mothur (M+D+M) or the ESPRIT programs as described in Materials and methods.
