# Supplementary material for: Potential Accumulative Effect of the Herbicide Glyphosate on Glyphosate-Tolerant Maize Rhizobacterial Communities over a Three-Year Cultivation Period
Source: PLoS One. 2011 Nov 11;6(11):e27558. doi: 10.1371/journal.pone.0027558 (PMC3214082; doi:10.1371/journal.pone.0027558)
Supplement: Table S4 — Similarity-based OTUs and species richness estimates at a 3%, 5% and 10% dissimilarity level for the samples from 2009. The species richness estimates were determined by using the MUSCLE, DNADIST and Mothur (M+D+M) combination or the ESPRIT program, as described in Materials and Methods. (PDF) [file pone.0027558.s005.pdf]

Table S2.3. Similarity-based OTUs and species richness estimates at a 3%, 5% and 10% dissimilarity level for the samples from 2009

**Field 1**	**2009**											
	**First sampling time**											
	**Untreated**						**Glyphosate**					
	**M+D+M**			**ESPRIT**			**M+D+M**			**ESPRIT**		
	3%	5%	10%	3%	5%	10%	3%	5%	10%	3%	5%	10%
**OTUs**	756	661	463	622	497	279	734	609	402	621	473	242
**ACE**	2794±544	1913±324	777±90	1611	1030	376	2579±465	1444±219	589±61	1552	980	284
**Chao1**	2583±471	1774±299	692±78	1412±216	969±152	369±47	2447±450	1251±175	544±56	1525±254	952±160	275±23
	**Final sampling time**											
	**Untreated**						**Glyphosate**					
	**M+D+M**			**ESPRIT**			**M+D+M**			**ESPRIT**		
	3%	5%	10%	3%	5%	10%	3%	5%	10%	3%	5%	10%
**OTUs**	753	639	401	589	460	255	703	575	345	582	461	236
**ACE**	2481±463	1481±223	573±57	1264	885	348	1779±280	1090±134	472±46	1004	748	271
**Chao1**	2279±381	1338±186	554±61	1271±197	806±117	340±46	1628±232	1003±123	460±52	923±98	680±75	264±21

**Field 2**	**2009**											
	**First sampling time**											
	**Untreated**						**Glyphosate**					
	**M+D+M**			**ESPRIT**			**M+D+M**			**ESPRIT**		
	3%	5%	10%	3%	5%	10%	3%	5%	10%	3%	5%	10%
**OTUs**	699	586	380	624	508	272	761	647	408	634	506	283
**ACE**	2222±410	1371±198	584±67	1551	1115	349	2526±452	1761±282	624±69	1344	1016	388
**Chao1**	1870±301	1233±184	543±65	1408±214	932±131	339±37	2278±376	1670±280	609±76	1323±187	951±141	400±61
	**Final sampling time**											
	**Untreated**						**Glyphosate**					
	**M+D+M**			**ESPRIT**			**M+D+M**			**ESPRIT**		
	3%	5%	10%	3%	5%	10%	3%	5%	10%	3%	5%	10%
**OTUs**	642	527	333	533	410	223	674	547	350	543	393	180
**ACE**	1719±282	1072±146	469±49	1072	699	275	2120±397	1262±185	497±52	1324	673	196
**Chao1**	1476±223	964±133	466±60	900±110	617±76	261±25	1993±356	1149±180	483±58	1241±212	648±95	187±9

The species richness estimates were determined by using the combination MUSCLE, DNADIST and Mothur (M+D+M) or the ESPRIT programs as described in Materials and methods.
