# Supplementary material for: Potential Accumulative Effect of the Herbicide Glyphosate on Glyphosate-Tolerant Maize Rhizobacterial Communities over a Three-Year Cultivation Period
Source: PLoS One. 2011 Nov 11;6(11):e27558. doi: 10.1371/journal.pone.0027558 (PMC3214082; doi:10.1371/journal.pone.0027558)
Supplement: Table S5 — Multiplex identifiers (MIDs). The Multiplex identifiers (MIDs) used for pyrosequencing the different samples are shown. (PDF) [file pone.0027558.s006.pdf]

Table S3. Roche Multiplex identifiers (MIDs)

Year	Sampling time	Field	Treatment	MID sequence	Reads
2007	First	1	Untreated	5′- CGTGTCTCTA -3′	8000
			Glyphosate-treated	5′- CTCGCGTGTC -3′	5876
		2	Untreated	5′- TCTCTATGCG -3′	13967
			Glyphosate-treated	5′- TGATACGTCT -3′	10561
	Final	1	Untreated	5′- CATAGTAGTG -3′	13606
			Glyphosate-treated	5′- CGAGAGATAC -3′	15477
		2	Untreated	5′- ACGCTCGACA -3′	39293
			Glyphosate-treated	5′- AGACGCACTC -3′	38052
2008	First	1	Untreated	5′- ATCAGACACG -3′	4946
			Glyphosate-treated	5′- ATATCGCGAG -3′	8725
		2	Untreated	5′- CTCGCGTGTC -3′	7361
			Glyphosate-treated	5′- TAGTATCAGC -3′	20146
	Final	1	Untreated	5′- TGATACGTCT -3′	11170
			Glyphosate-treated	5′- TACTGAGCTA -3′	3429
		2	Untreated	5′- CGAGAGATAC -3′	10964
			Glyphosate-treated	5′- ACGAGTGCGT -3′	15457
2009	First	1	Untreated	5′- AGACGCACTC -3′	16732
			Glyphosate-treated	5′- AGCACTGTAG -3′	4893
		2	Untreated	5′- ATATCGCGAG -3′	9300
			Glyphosate-treated	5′- CGTGTCTCTA -3′	5170
	Final	1	Untreated	5′- TAGTATCAGC -3′	6399
			Glyphosate-treated	5′- TCTCTATGCG -3′	9916
		2	Untreated	5′- TACTGAGCTA -3′	36103
			Glyphosate-treated	5′- CATAGTAGTG -3′	6186

The Multiplex identifiers (MIDs) used for pyrosequencing of the different samples are shown.
